# Two Cases of Pulmonary Tuberculosis Caused by Mycobacterium tuberculosis subsp. canetti

**DOI:** 10.3201/eid0811.020017

**Published:** 2002-11

**Authors:** Jean Miltgen, Marc Morillon, Jean-Louis Koeck, Anne Varnerot, Jean-François Briant, Gilbert Nguyen, Denis Verrot, Daniel Bonnet, Véronique Vincent

**Affiliations:** * Hôpital d’instruction des armées Laveran, Marseille, France; † Hôpital d’instruction des armées Val de Grâce, Paris, France; ‡ Institut Pasteur, Paris, France

## Abstract

We identified an unusual strain of mycobacteria from two patients with pulmonary tuberculosis by its smooth, glossy morphotype and, primarily, its genotypic characteristics. Spoligotyping and restriction fragment length polymorphism typing were carried out with the insertion sequence IS6110 patterns. All known cases of tuberculosis caused by Mycobacterium canetti have been contracted in the Horn of Africa.

The Mycobacterium tuberculosis complex includes the following mycobacteria, which are characterized by a slow growing rate: M. tuberculosis, M. africanum, M. bovis, and M. microti (1). In recently published reports of two cases of lymphatic node tuberculosis (TB), the strains were recognized as belonging to a new taxon of M. tuberculosis (2,3). These isolates were characterized by a highly particular growing pattern, and the colonies appeared smooth and glossy. A complete genetic study of these strains led to their integration into the M. tuberculosis complex. This strain, identified as M. tuberculosis subsp. canetti or, more simply, M. canetti, was first isolated in 1969 by Georges Canetti from a French farmer. The strain was preserved at the Pasteur Institute where its antigenic pattern was studied extensively. We report two cases of pulmonary TB caused by this strain. The two patients had also lived in East Africa.

## The Study

### Patient 1

In September 1998, a 36-year-old male soldier in the French Foreign Legion with hemoptysis was sent back to France from Djibouti. He expectorated bloody sputum after running and on a few other occasions. His medical history was not unusual. When the patient was hospitalized, 2 weeks after the initial symptoms, he began to experience progressive fatigue. He did not experience fever, weight loss, night sweats, anorexia, cough, dyspnea, or chest pain, and did not produce sputum.

Results of the clinical examination were normal. The Mantoux test, performed with 10 IU of purified tuberculin (Aventis-Pasteur-MSD, Lyon, France), yielded a maximum transverse diameter of induration of 15 mm. Laboratory values were normal (Table). The chest X-ray showed a triangular consolidation of the left upper lobe with blurred limits and small cavitary lesions. No other contiguous mediastinohilar anomalies were visible. A computed tomographic scan confirmed the cavitary syndrome: three excavated nodular images showed radiating spicules within a micronodular infiltrate. Bronchoscopy showed a moderate inflammation of airway mucosa, especially in the left upper lobe. Biopsy specimens exhibited nonspecific inflammation.

**Table:** Laboratory values for both patients infected with Mycobacterium tuberculosis subsp. canetti

Laboratory test	Patient 1	Patient 2
Sedimentation rate (mm)	3	3
C-reactive protein (mg/L)	7.3	4.28
Fibrinogen (g/L)	3.64	6.3
Blood count		
Hemoglobin (g/dL)	16.8	14.1
Platelets (x109/L)	194	274
White cells (x109/L)	10.10	9.54
Neutrophils (%)	66.2	69.4
Eosinophils (%)	2	8.6
Lymphocytes (%)	21.3	17.2
Basophils (%)	0.6	0.9
Monocytes (%)	9.9	3.9
Aspartate aminotransferase (U/L)	21	16
Alanine aminotransferase (U/L)	19	23
Creatinine (µmol/L)	89	97
Glucose (mmol/L)	4.7	4.4

A bronchial washing smear from the left upper lobe was positive for acid-fast bacilli. Serologic tests for HIV-1 and HIV-2 were negative. No evidence of disease was found elsewhere; the patient did not experience bone pain. Results of neurologic and ophthalmologic examinations were normal; no lymphadenopathy or hepatosplenomegaly were found and the genitalia were normal. Auscultation revealed no pericardial fremitus; no ascitic fluid was detected. The urinary sediment contained <1,000 red blood cells/L and <5,000 leukocytes/L. Antituberculosis chemotherapy was begun with four drugs: rifampicin, isoniazid, ethambutol, and pyrazinamide. Cultures revealed a strain identified as M. tuberculosis subsp. canetti that was susceptible to all primary antituberculous drugs. Therefore, rifampicin and isoniazid were continued for 3 more months for a total treatment period of 6 months. The patient’s response to treatment was favorable, and he remained asymptomatic.

### Patient 2

A 55-year-old male soldier in the French Foreign Legion, who returned from Djibouti, was hospitalized in September 1999 after his chest x-ray showed abnormal findings. He was a nurse and had been occasionally in charge at the Djibouti Hospital for 2 years. His medical history was unremarkable. Eight months before he returned to France, he experienced asthenia, anorexia, and a weight loss of 3 kg. The symptoms resolved spontaneously after 2 months, and he had been asymptomatic since then. He had no history of cough, sputum production, hemoptysis, dyspnea, fever, or night sweats.

Results of a clinical examination and of laboratory studies were normal (Table), except for hypereosinophilia. Serologic tests for schistosomiasis, hydatidosis, distomiasis, amebiasis, toxocariasis, and trichinosis were negative, and parasites were not found in stool samples. Thoracic radiographs performed when he came back from Djibouti showed parenchymal consolidation of the right upper lobe with small cavities. Sputum was not produced. A gastric aspirate smear was negative for acid-fast bacilli, and a bronchial aspiration smear was positive for acid-fast bacilli. HIV serology was negative, and no other site of the infection was found. Drug therapy was initiated with rifampicin, isoniazid, ethambutol, and pyrazinamide for 2 months. Cultures of bronchial aspirates were positive within 14 days; later, cultures of two gastric aspirates were positive for acid-fast bacilli. An M. tuberculosis subsp. canetti isolate was identified, which was susceptible to all primary antituberculous drugs. The treatment was then extended for 4 months with rifampicin and isoniazid. The patient's response to treatment was favorable.

The following methods were used to identify the etiologic agent. First, the samples were decontaminated with N-acetyl-L-cysteine/NaOH. Acid-fast bacilli were detected by auramine staining, the positive smears also were stained with Ziehl-Nielsen stain. The samples were then seeded onto Löwenstein-Jensen and Coletsos slants and also into a liquid system, the BBL Mycobacterial Growth Indicator Tube (MGIT, BD Diagnostic Systems, Sparks, MD).

The mycobacteria were identified by using a specific DNA probe (Gen-Probe, Gen-Probe Incorporated, San Diego, CA) and by performing the usual biochemical tests (nitrate reduction, 68°C catalase resistance, niacin production).

The Pasteur Institute of Paris used two methods for typing: restriction fragment length polymorphism (RFLP) analysis and spoligotyping. In RFLP analysis, after digestion of the M. tuberculosis strain's genomic DNA with PvuII restriction enzyme and agarose gel migration, the DNA was transferred on a membrane, according to the Southern method, and then hybridized with an insertion sequence IS6110 probe (4). In the spoligotyping method, after DNA direct repeat amplification, the labeled polymerase chain reaction product was used as a probe to hybridize with 43 synthetic spacer oligonucleotides (DNA sequences derived from the direct repeat [DR] region of M. tuberculosis, H37Rv and M. bovis BCG P3), which were attached to a carrier membrane (5). The sensitivity to antituberculous drugs was determined by the indirect proportion method.

**Figure 1:**
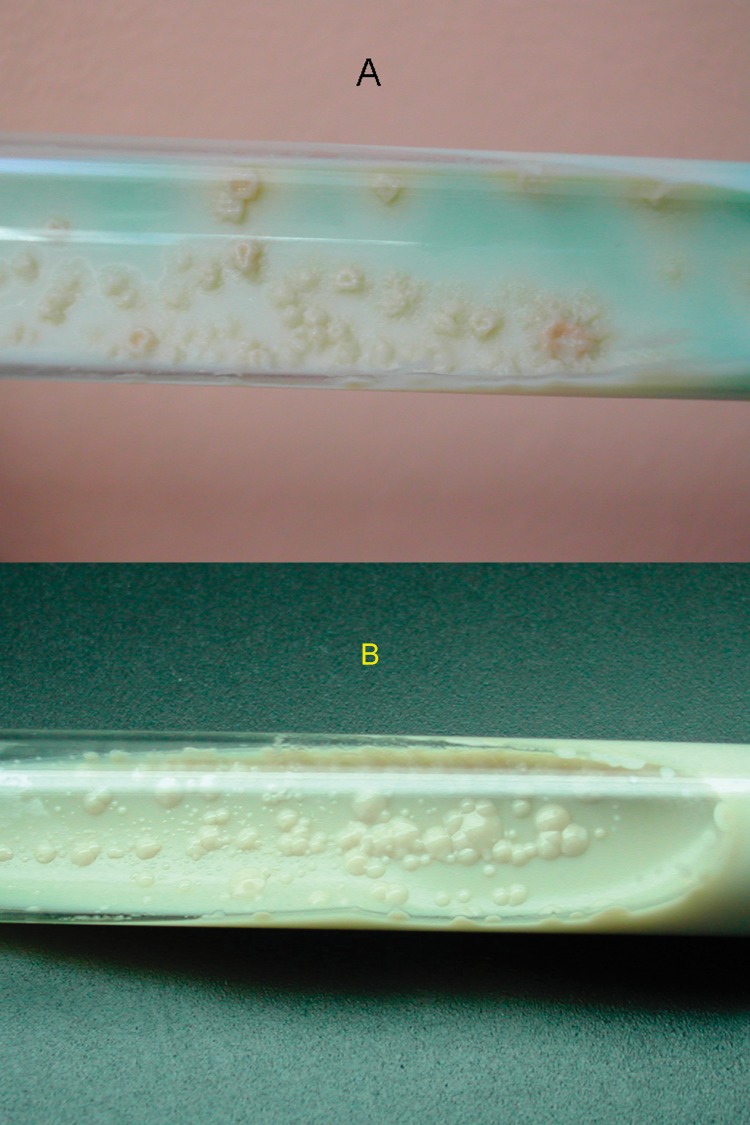
Colony morphology on Löwenstein-Jensen slants, showing M. canetti and M. tuberculosis strains. (A) Colonies of M. tuberculosis are rough, thick, wrinkled, have an irregular margin, and are faintly buff-colored. (B) M. canetti exhibits smooth, white and glossy colonies.

MGIT results were positive for the two cultures in 9 and 12 days, respectively. On Löwenstein-Jensen slants, the cultures were positive in 12 and 14 days, respectively. The white, smooth, and glossy colonies were characteristic of M. tuberculosis subsp. canetti (Figure 1). The two strains had the same phenotypic and genotypic pattern; 68°C catalase was negative, and they reduced nitrate, as do other M. tuberculosis species, but they did not produce niacin. The DNA probe, Gen-Probe, confirmed that these strains belonged to the M. tuberculosis complex.

**Figure 2:**
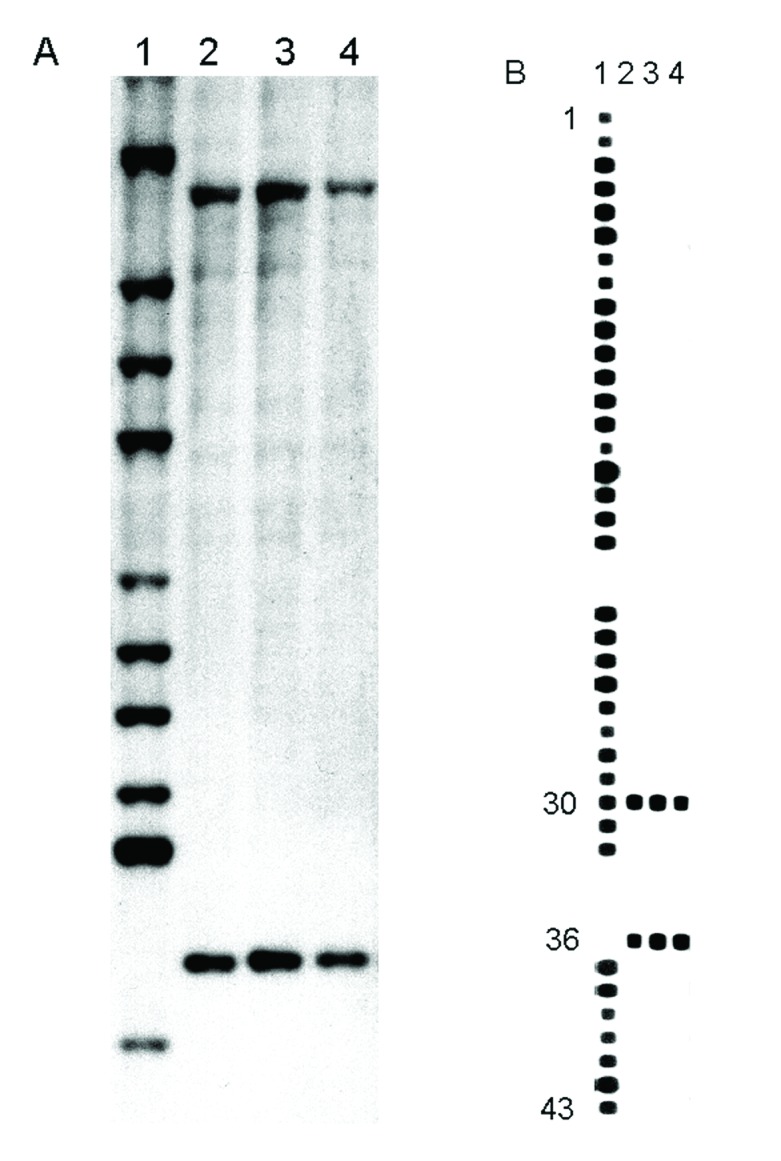
(A) IS6110 hybridization patterns of PvuII-digested genomic DNA. Lane 1, Mycobacterium tuberculosis Mt 14323 (reference strain). Lane 2, M. canetti strain NZM 217/94. Lanes 3 and 4, the strains isolated from French legionnaires with pulmonary tuberculosis (TB). (B) Spoligotyping patterns. Lane 1, M. tuberculosis H37Rv (reference strain). Lane 2, M. canetti strain NZM 217/94. Lanes 3 and 4, the strains isolated from French legionnaires with pulmonary TB.

These strains contained two copies of IS6110. Spoligotyping showed that they shared only 2 of the 43 oligonucleotides reproducing the spacer DNA sequences of M. tuberculosis, H37Rv and M. bovis BCG P3. This profile is characteristic of M. tuberculosis subsp. canetti (Figure 2).

## Conclusion

In 1997, van Soolingen reported a case of lymph node TB in a 2-year-old Somali child on the child’s arrival in the Netherlands in 1993 (2). In 1998, Pfyffer described abdominal lymphatic TB in a 56-year-old Swiss man (who lived in Kenya) with stage C2 HIV infection (3). These strains of M. canetti (So93 from the Somali child and NZM 217/94 from the Swiss man) have been studied extensively. In culture they grow faster than other strains in the M. tuberculosis complex. The So93 strain expands by one rough colony for every 500 smooth colonies. They appear smooth, white, and glossy because of the high amount of lipooligosaccharides in the membrane (6); the So93 rough colonies lack this amount (2).

Two copies of the IS6110 insertion sequence were found in the NZM 217/94 and So93 genome. This fingerprint matched none of the 5,000 other strains preserved in the laboratory of van Soolingen (Bilthoven, the Netherlands) (2). The strains we observed also showed two copies of IS6110.

So93, NZM 217/94, and our two strains share only 2 of 43 identical repeated sequences that have been observed by spoligotyping. Study of the IS6110 RFLP patterns and of the spacer DNA sequences of the DR locus confirmed that M. tuberculosis, M. bovis, M. africanum, M. microti, and M. canetti represent a closely related group of mycobacteria that are clearly distinct from other mycobacterial species. In the M. tuberculosis complex, M. canetti appears to be the most divergent strain (2).

We believe that this is the first published report of pulmonary disease caused by M. canetti. Our two cases confirm that M. canetti is able to involve lungs, like any other other member of the M. tuberculosis complex and is able to affect immunocompetent subjects. The clinical features of these two pulmonary cases of TB caused by M. canetti are not specific.

TB caused by M. canetti appears to be an emerging disease in the Horn of Africa. A history of a visit to the region should cause this strain to be considered promptly. As travel to this area becomes more frequent, and mycobacterial identification techniques improve, the number of diagnosed cases will likely increase.
